# Three discipline collaborative radiation therapy (3DCRT) special debate: Systemic radiotherapy using targeted isotopes is the best hope for advancing curative radiation therapy

**DOI:** 10.1002/acm2.14533

**Published:** 2024-10-24

**Authors:** Bridget F. Koontz, Marianne Koritzinsky, Jacqueline E. Zoberi, Stephen L. Brown, Xuanfeng Ding, Jeffrey Wong, Michael C. Joiner, Michael M. Dominello, Jay Burmeister

**Affiliations:** ^1^ Department of Radiation Oncology AdventHealth Cancer Institute Orlando Florida USA; ^2^ Princess Margaret Cancer Centre, University Health Network / Department of Radiation Oncology University of Toronto Toronto Ontario Canada; ^3^ Department of Radiation Oncology Washington University School of Medicine Saint Louis Missouri USA; ^4^ Department of Radiation Oncology, Henry Ford Health Michigan State University Health Sciences Detroit Michigan USA; ^5^ Department of Radiation Oncology William Beaumont University Hospital, Corewell Health Royal Oak Michigan USA; ^6^ Department of Radiation Oncology City of Hope Cancer Center Duarte California USA; ^7^ Department of Oncology Wayne State University School of Medicine Detroit Michigan USA; ^8^ Gershenson Radiation Oncology Center Barbara Ann Karmanos Cancer Institute Detroit Michigan USA

**Keywords:** KEY WORDS, radiopharmaceutical therapy, RPT, systemic radiation therapy

## 1 THREE DISCIPLINE COLLABORATIVE RADIATION THERAPY (3DCRT) DEBATE SERIES

Radiation oncology is a highly multidisciplinary medical specialty, drawing significantly from three scientific disciplines—medicine, physics, and biology. As a result, discussion of controversies or changes in practice within radiation oncology involves input from all three disciplines. We have adopted a similar “team‐science” approach to the traditional debates featured in this journal. This article is part of a series of special debates entitled “Three Discipline Collaborative Radiation Therapy (3DCRT)” in which each debate team includes three multidisciplinary team members with the hope that this format will be both engaging for the readership and foster further collaboration across the science and clinical practice of radiation oncology.

## 2 INTRODUCTION

Radiation oncology has witnessed a technical revolution over the past several decades. The advent of advanced imaging and delivery techniques has dramatically improved our ability to target tumors with radiotherapy. However, local control of gross disease does not necessarily result in long‐term success as a result of locoregional and/or distant microscopic disease. Traditional external beam and brachytherapy techniques are designed to eradicate disease and spare normal tissues through geometrical targeting. In contrast, systemic therapies are targeted on the cellular level, are not constrained to specific treatment locations or even by our ability to locate disease, and are therefore capable of eradicating tumor cells throughout the body. Systemic therapies are not new, having been around in some form for the better part of a century. However, the recent development and relative success of several new systemic therapies has breathed new life into this treatment technique, and it has rapidly become one of the most exciting areas of research and development in radiation oncology. How far can we hope to extend the application and capabilities of systemic therapy? Will emerging cellular delivery mechanisms, new imaging techniques, and the use of alpha emitting isotopes revolutionize systemic therapy? Can we expect a future in which systemic therapy provides the best hope for cure for a large number of disease sites, or will it continue to be limited to a relatively small number of unique treatment applications? This is the topic of the current 3DCRT debate.

Arguing for the proposition will be Drs Bridget Koontz, Marianne Koritzinsky, and Jacqueline Zoberi. Dr Koontz trained at Harvard Medical School and Duke University School of Medicine. She was a faculty member in the Duke University Department of Radiation Oncology for 14 years before serving as US Chief Medical Officer for GenesisCare from 2021 to 2023. Dr Koontz is currently the Medical Director of Radiation Oncology services at the AdventHealth Cancer Institute in Orlando, Florida. She also is an Affiliate Professor at East Carolina University and a Fellow of the American Society for Radiation Oncology. Dr Koritzinsky has a PhD in radiobiology from the University of Oslo (Norway) and completed postdoctoral training at the University of Maastricht (The Netherlands). She is a Senior Scientist at the Princess Margaret Cancer Centre in Toronto (Canada). The aim of her research program is to increase our understanding of molecular and cellular responses to altered metabolism in the tumor microenvironment. Dr Zoberi earned a PhD in medical physics from the University of Chicago and completed physics residency training at Washington University in Saint Louis, where currently she is a Professor of Radiation Oncology and Chief of Brachytherapy Physics. She is actively involved in multiple initiatives within AAPM, ASTRO, and ABS focused on brachytherapy, radiopharmaceutical therapy (RPT), and medical physics education.

Arguing against the proposition will be Drs Stephen Brown, Xuanfeng Ding, and Jeffrey Wong. Dr Brown is a Senior Scientist for the Department of Radiation Oncology at Henry Ford Health (HFH), Detroit, Michigan; Professor of Radiology, Michigan State University, East Lansing, Michigan; Professor of Oncology, Wayne State University, Michigan; and co‐chair of Translational Oncology Research, Henry Ford Cancer Institute, HFH. His research focuses on early imaging predictors of tumor response, radiosensitizers, and minimizing normal tissue radiation injury. Dr Ding is Associate Professor and lead proton physicist at the proton therapy center at William Beaumont University Hospital, Corewell Health, Michigan. Dr Ding's research interests include proton arc technique, adaptive therapy, and motion management. In 2024, he received the John Laughlin Early‐Career Scientist Award from the AAPM. He is past president of the Great Lakes Chapter of the AAPM. Dr Wong is a Professor in the Department of Radiation Oncology and the Department of Immunology and Theranostics at City of Hope in Los Angeles, California. His primary areas of research include the development of novel targeted radiopharmaceuticals for therapy of non‐hematopoietic and hematopoietic malignancies and the development of targeted total marrow irradiation for bone marrow transplantation.

## 3 OPENING STATEMENTS

### 3.1 Bridget F. Koontz, MD; Marianne Koritzinsky, PhD; Jacqueline E. Zoberi, PhD

The field of radiation oncology has seen much progress over the decades, the majority of which has involved technical advances in external beam and brachytherapy delivery; a few examples include CT‐based planning for both external beam and HDR, IMRT, and MR linacs. There have been biological advances, too, some paradigm‐shifting (e.g., alpha/beta modeling) and some still being developed (e.g., radioimmunology). So, what are the current challenges that limit our ability to “advance curative radiation therapy?” They are three‐fold: microscopic disease, radioresistance, and harnessing the power of the immune system. Targeted radioisotope therapy, hereafter referred to as radiopharmaceutical therapy or RPT, represents our best hope to overcome these limitations.

Current radiotherapy delivery methods excel at local control. The technical advances are leveraged to provide real‐time visualization of a target structure and provide a lethal dose to the tumor while avoiding critical organs at risk.^1^, ^2^ However, the contribution of image guidance to cure becomes incrementally smaller, as cure is limited by locoregional or distant microscopic disease. Systemic therapies that can be absorbed by individual cancer cells anywhere in the body will be necessary to eradicate disseminated and metastasized cells. RPT is the only radiation therapy modality that offers the unique capacity of targeting radiation based on cellular characteristics rather than anatomic location. This can be realized by radioisotopes that naturally home in on certain cell types (e.g., I‐131 to thyroid and Ra‐223 to bone), or radioisotopes conjugated to ligands that leverage receptor specificity (e.g., Lu‐177‐PSMA ligand to PSMA‐expressing prostate cancer cells, and Lu‐177‐dotatate to somatostatin‐receptor positive neuroendocrine cancer cells). Numerous other targeting strategies are under development, aligned with the clear vision that in order to cure more cancer patients with radiotherapy, we need to both control local and distant disease.

Cancer cells can also have inherent or adaptive radioresistance, which must be overcome for radiotherapy to be curative. Although heavy ion modalities and hypoxia modulation represent possible approaches, these options have been around for decades without penetrating daily clinical practice due to high cost, patient discomfort, and marginal benefit. Concurrent chemotherapies are likely already employed to their potential, and blocking radiation resistance pathways are for the most part not curative and often associated with a prohibitively small therapeutic window. In contrast, there is considerable excitement around the ability of RPT to overcome radioresistance through delivering alpha particle therapy (e.g., Ra‐223 for bone metastasis and Ac‐225‐PSMA for prostate cancer). In addition, RPT can also be used to boost effective tumor dose in combination with conventional radiotherapy, addressing radioresistance while keeping toxicity acceptable.

Immense excitement is associated with the promise of harnessing immune cell interactions to potentiate radiotherapy. Radiotherapy is inherently immunogenic, stimulating the release of antigens that prime dendritic cells and activate cytotoxic T‐lymphocytes. However, immune cells are also radiosensitive, and the potential mitigation of circulating immune cell viability through the irradiation of substantial blood volumes during external beam radiation can be a concern. Furthermore, radiotherapy fields frequently include tumor‐draining lymph nodes that are essential hubs for immune cell maturation. This has been clearly shown to dampen anti‐tumor immune responses.^3^ With its targeted strategy, RPTs are predicted to circumvent these immune‐suppressive aspects of traditional radiotherapy. In addition, low‐dose targeted radionuclide therapy was demonstrated to switch immunologically suppressed tumors to be responsive to immune checkpoint blockade.^4^ As such, RPT may offer a double advantage through both alleviating immune suppression and potentiating immune activation.

While RPT is the best hope for advancing curative radiation therapy, development is needed to optimize this treatment approach. More mechanistic insight into which patients’ benefit is necessary, along with predictive biomarkers for patient selection. Research in personalized dosimetry for RPT is evaluating how to determine appropriate activity levels, infusion schedules, and dose‐limiting toxicities, based on isotope and organ at risk. There is debate about how to best find the appropriate dosing regimen for new RPT agents,^5^ and employment of classical radiobiology frameworks (e.g., alpha/beta modeling, proliferation and repair factors, and dose response‐relationships) are lagging behind. Cost is a major barrier to access that will need to be addressed at a system level in step with policy on health care costs and equity.

RPT is in its infancy compared to other radiotherapy modalities, with only a few cancers having FDA‐approved RPT therapies. Exponential growth from academic and industry research and development will continue to create more diverse and better agents, providing options for a wider variety of cancer types and continued improvement in a targeting ligand's ability to deliver “precision” systemic radiotherapy. This will no doubt propel this field forward in the coming decades, to meet the increasing demand for the development of RPT agents.^6^

In summary, current external beam RT and brachytherapy methods already provide excellent local control for many cancers. However, eradication of measurable localized tumor deposits is only part of the broader picture for many cancer types. A systemic approach which also mitigates radioresistance and exploits the power of the immune system, like RPT, is required to take the next leap and provide even greater cure rates using radiotherapy.

### 3.2 Stephen Brown, PhD; Xuanfeng Ding, PhD; Jeffrey Wong, MD

Arguing against the proposition, we submit that systemic radiotherapy using targeted isotopes is a promising therapeutic approach against cancer but at present is limited in both theory and practice. From clinical, radiobiological, and medical physics perspectives, the systemic use of targeted isotopes does not represent the best hope for advancing curative radiation therapy.

Systemic radiotherapy using isotopes that target physiological mechanisms specific to neoplasia^7^ is a subset of RPT and is not new. RPT has been used for compassionate care for decades without a curative intent. Fundamentally, RPT relies on α‐ or β‐particle‐emitting agents, a principle that has not changed in 40 years, despite shifting focus from single isotopes to a broader range of radionuclides.^8^ The targeted approach holds promise if reported efficacy and relatively low toxicity compared to traditional chemotherapy can be reproduced.^9^ However, despite its decades‐long history, the technology is in its infancy and challenges remain.

Clinical challenges include logistical limitations including the cost and availability of isotopes. Who is responsible for treatment (radiation oncologists or nuclear medicine physicians) and is reimbursement sufficient (arguably the practical issue that put hyperthermia utilization on the back burner)? Perhaps the radiation oncology departments are best suited for dosimetry and treatment planning issues, but intravenously administered agents are not routine procedures in radiation oncology clinics. How best to combine local or regional radiotherapy of primary tumors with radiopharmaceuticals for metastatic disease is not resolved.

Radiobiology challenges include the limited specificity of targeted therapy and off‐target effects. How many administrations (fractions) is ideal is not known. The field is hampered by the lack of a standard animal model available. Animals are limited by differences in biology, physiology, and size from the humans they strive to model.

Physics challenges include delivery challenges since distances are greater between source and community hospitals in the US than the initial successful studies performed in Europe. The issue of radiation dosimetry has not been resolved. Does every patient receive the same dose of isotope or does the dose of isotope depend on tumor size?

The critical question is whether the newer isotopes, with varying half‐lives or x‐ray emissions for dosimetric quality assurance, will improve therapeutic outcomes. The answer, considering the unchanged underlying physics, is likely no. What has revived the interest is the growing investment from pharmaceutical companies^10^ and increasing use and investigation of small‐molecule radiotherapeutics targeting receptors such as PSMA receptor and folate receptor (FR),^11^, ^12^, ^13^ which have increased retention time and dose delivered to tumors. Recent interest is also from PSA responses in prostate cancer, a relatively radiosensitive solid tumor, but measurable responses remain infrequent. The key to meaningful improvement lies in revolutionary targeting techniques that could address a broader spectrum of cancers beyond thyroid,^14^ hematological,^15^ hepatic,^16^ and prostate cancers.^11^ However, the targeting criteria for delivering radionuclides remain limited, with only around 10 specific targets in clinical use or investigation out of over 200 cancer types.^7^ As monotherapy, targeted RPT in a macroscopic disease setting is currently not curative and will likely also be the case in the microscopic disease setting given that hematopoietic toxicity is usually dose‐limiting. RPT's “best hope for advancing curative radiation therapy” may be in the multi‐modality setting if it can be added without significant additional toxicities. Long‐term toxicities need to be better characterized, especially for the recently introduced alpha‐emitters, before widespread application to potentially curative patients.

Similar to external radiotherapy, dosimetric quality is crucial in RPT, as it determines treatment outcomes like tumor control probability (TCP) and normal tissue complication probability (NTCP) with the goal of maximizing radiation to tumors while minimizing exposure to healthy tissues.^17^ Accurate dose distribution in planning and delivery is essential for predicting therapeutic success.^18^, ^19^ Unlike external beam radiotherapy, where extensive clinical outcome data supports TCP and NTCP models, RPT dosimetry is still in its infancy. Current RPT dosimetry often uses anatomical geometries representing average patients rather than specific individuals. While recent advancements in image‐based, patient‐specific dosimetry offer more accurate dose distributions, there are still significant challenges. Because RPT uses localized α‐particle or β‐particle radiation, organ toxicity might not correlate with whole‐organ absorbed doses, as in external radiotherapy, but rather with “hot spots” in organ subregions.^7^, ^20^, ^21^ Unfortunately, existing imaging techniques lack the resolution to precisely identify these microscopic distributions.^7^ Additionally, a new radiobiology model is essential for broader clinical implementation, aiming to cure cancers while minimizing toxicity.

Finally, we note the cautionary tales of Bexxar and Zevalin. These two RPT agents were shown to be effective but are no longer available. It is obvious that we need to learn the lessons from past experiences.^22^

In summary, while systemic radiotherapy with targeted isotopes holds promise, it faces significant hurdles in terms of targeting capabilities, dosimetric accuracy, and radiobiological understanding. Broad adoption will require a more comprehensive multidisciplinary approach, updated radiobiological models, and improved imaging and targeting techniques.

## 4 REBUTTAL

### 4.1 Bridget F. Koontz, MD; Marianne Koritzinsky, PhD; Jacqueline E. Zoberi, PhD

We appreciate our colleagues' comments regarding RPT being in the early development phase as a technology. This renders most of the concerns raised as surmountable challenges, which the field is positioned to solve.

We need to move away from the notion that the infrastructure for RPT needs to be within nuclear medicine or radiation oncology, and instead view RPT as an opportunity for growth and collaboration between both specialties. From a regulatory perspective, physicians who have completed training in nuclear medicine or radiation oncology are eligible to become authorized users (AUs) for oral or parenteral administration of therapeutic unsealed sources. Nuclear medicine physicians are essential for conducting and interpreting diagnostic imaging studies, as is the case for theranostic agents. Radiation oncologists play a key role in longitudinal patient care, and are well‐positioned to lead combined modality radiation therapy with RPT. Thus, a patient‐centered, multi‐disciplinary approach should be the focus, rather than ownership by one specialty over another, to advance the potential of RPT.^23^

A valid point is raised around cost and access, but efforts to contain the high costs of medical care are underway within the pharmaceutical industry and government, which should provide a framework for the field to address this within RPT. Initiatives are underway by health policy groups to advocate for more effective payment models in radiation oncology, and strategies have been proposed to address reimbursement.^24^ Access needs to be addressed by manufacturers, and improvements in diagnostic PET agents show there is an ability to do so.

The need for better radiobiological modeling and understanding of dose–response relationships is becoming well‐recognized in the field. Clinical data clearly demonstrate therapeutic benefit of dose escalation based on personalized dosimetry and suggest that patients may often be under‐dosed today.^25^ Personalized dosimetry for all patients will be the catalyst for change and is currently being implemented in some countries. An EU directive states that “target volumes shall be individually planned and their delivery appropriately verified.” Accordingly, the European Association of Nuclear Medicine proposes three levels of compliance with this directive, aligning with principles defined by the International Commission on Radiation Units and Measurements (ICRU),^26^ ranging from activity‐based and patient‐averaged dosimetry for all patients to dosimetry‐guided patient‐specific prescription and verification for patients on research studies. Similar, and potentially expanded, recommendations from governing bodies elsewhere in the world will help propel this field forward.

Two points we would counter: That radioisotope therapy does not have the potential to cure, and that retracted drugs prove failure of a strategy. The therapeutic challenge is to deliver sufficient dose without causing toxicity. For all molecular‐targeted agents, success relies on the specificity of the ligand‐target interaction. This has not hampered the approval of targeted drugs for cancer treatment. At least 124 new drugs for 374 cancer indications were FDA approved between 2003 and 2021.^27^ Specificity and pharmaco‐kinetics and pharmaco‐dynamics can also be tuned with new biomolecular scaffolds under development, such as bi‐functional antibodies or fragments (diabodies, nanobodies, etc.). Animal models have significant limitations and will not be able to answer all questions of fractionation and dose,^28^ requiring clinical studies. As development of targeting agents and radioisotope ligands proceeds (i.e., alpha emitters), we anticipate improvement in the therapeutic window for more cancers. As for Bexxar and Zevalin, these were effective, but other treatment combinations were comparatively tested and were superior.

Exciting times lie ahead for RPT, with new drugs and combinations built on personalized dosimetry and radiobiological modeling, with mindfulness of cost and access.

### 4.2 Stephen Brown, PhD; Xuanfeng Ding, PhD; Jeffrey Wong, MD

Our esteemed colleagues present powerful arguments supporting the perspective that we were tasked to defend that systemic radiotherapy using targeted isotopes is fraught with problems that indicate it is not the best hope for advancing curative radiation therapy.

Their opening statement thoughtfully outlines the limitations of RPT that have remained unchanged for decades. They state that more mechanistic insight into which patients would benefit and more effective predictive biomarkers for patient selection are needed. They assert personalized dosimetry is needed to determine appropriate activity levels, infusion schedules, and dose‐limiting toxicities based on isotope choice and organs at risk. Other challenges they describe include the appropriate dosing regimen for RPT agents, the appropriate employment of classical radiobiology frameworks (e.g., alpha/beta modeling, proliferation and repair factors, and dose–response relationships) and cost considerations. As our colleagues correctly summarize, RPT is in its infancy compared to other radiotherapy modalities.

Additionally, they identified three areas that challenge our ability to advance curative radiation therapy: microscopic disease, radioresistance, and harnessing the power of the immune system. In response, we contend that eliminating microscopic disease does not necessitate targeting. Similarly, there are many potential avenues to overcome the challenge of radioresistance. Finally, there is no clinical evidence that RPT alleviates immune suppression.

What more mature modality is available “to advance the hope for advancing curative radiation therapy”? We believe one example is particle therapy. Level 1 evidence has been steadily building in favor of particle therapy for cancers such as oropharyngeal cancer,^29^ and ongoing randomized clinical trials show promise.^30^ Additionally, particle therapy has proven to be an effective solution for radioresistant tumors, as its high linear energy transfer (LET) and dose escalation are safe and effective.^31^, ^32^ Emerging clinical evidence supports combining particle therapy with immunotherapy. Therefore, numerous clinical trials are underway to examine the benefit of particle therapy against distant metastasis in addition to local control.^33^, ^34^, ^35^, ^36^, ^37^

Finally, effective agents such as Bexxar and Zevalin are no longer available due to the lack of interest from the majority of nuclear medicine and radiation oncology physicians in providing these services and a lack of accessibility of these agents, especially in the community setting.^38^ RPT as monotherapy will not likely be curative, and radiation oncologists will have a critical role in designing and leading trials to determine how best to integrate these therapies with other modalities, including external beam radiotherapy, brachytherapy, particle beam irradiation, radio‐sensitizing agents, immunotherapy, chemotherapy, and as conditioning agents in bone marrow transplantation. As multi‐modality therapy becomes more complex, the requirement to standardize dosimetry for patient‐specific dosing to optimize efficacy and minimize toxicity is inevitable. Increasing accessibility to agents is needed to realize the full potential of RPT. It is estimated that 100−300 new centers are needed,^39^ which cannot be easily accomplished by a single specialty, especially if limited to hospital‐based practices. The success or failure of RPT depends on practical issues such as availability, cost, and utilization. Based on recent history, RPT is not the best hope for advancing curative radiation therapy.

## AUTHOR CONTRIBUTIONS

The first six authors contributed equally to this work. All authors were responsible for preparation of arguments, and/or were involved in writing and reviewing the manuscript.

## CONFLICT OF INTEREST STATEMENT

Dr Koontz holds equity and leadership roles for Rythera Therapeutics and has served on advisory boards for Lantheus and Blue Earth Diagnostics.
